# Orbital volume changes during growth and development in human children assessed using cone beam computed tomography

**DOI:** 10.1186/s13005-022-00310-9

**Published:** 2022-02-28

**Authors:** Eric A. Smith, Caroline S. Halbach, Adriana Z. Robertson, Aden M. Peterson, Andrew R. Harrison, Thorsten Grünheid, Brent E. Larson, Ali Mokhtarzadeh

**Affiliations:** 1 Division of Orthodontics, University of Minnesota, Minneapolis, Minnesota United States; 2 Department of Ophthalmology & Visual Neurosciences, University of Minnesota, Minneapolis, Minnesota United States; 3 Faculty of Law & Rotman School of Management, University of Toronto, Toronto, Ontario Canada; 4 Department of Orthodontics, University of Iowa, Iowa City, Iowa United States

**Keywords:** Orbit, Craniofacial, Growth and development, Cone Beam Computed Tomography, CBCT, Segmentation, Human

## Abstract

**Objectives:**

To measure growth-related changes in orbital volume from childhood to the late teenage years using cone-beam computed tomography (CBCT) scans.

**Methods:**

This retrospective cohort study involved 65 (24 male, 41 female) healthy Caucasian children (ages 6–18 years) with existing serial craniofacial CBCT scans. CBCT scans were available for 292 orbits. Each orbit was transformed into a closed space with well-defined boundaries, and orbital volume was measured using manual segmentation. A novel statistical analysis was applied to extract the maximum amount of longitudinal information from the data. Intra- and inter-operator correlation coefficients were calculated from replications performed on a random subset of 10% of the sample.

**Results:**

Orbital volume increased at a rate of 1–2% annually until the late teenage years. Intra- and inter-operator agreement between repeated measurements were >90%.

**Conclusions:**

Orbital volume increases by 1–2% per year throughout childhood continuing until the late teenage years. This annual increase is large enough to be clinically relevant as it may lead to less-than-optimal long term surgical outcomes when reconstructive surgery for the pediatric anophthalmic socket is required.

**Supplementary Information:**

The online version contains supplementary material available at 10.1186/s13005-022-00310-9.

## Introduction

A number of genetic, developmental, and pathological conditions affect orbital development, often necessitating surgical intervention at an early age. Despite the development of techniques and materials aimed at improving outcomes of orbital implantation, foundational knowledge on the rate and timing of orbital growth in humans is scarce. Depending on the source, the orbit is reported to reach maturity between 3 and 18 years of age [1–6]. Studies attempting to model orbital volume changes in childhood have faced measurement and statistical challenges [5, 6]. The only conclusion that can be confidently drawn from the available literature is the broad generalization that orbital volume changes very slowly during childhood and adolescence, if at all.

Where regulation permits, orthodontists are increasingly using cone-beam computed tomography (CBCT) scans as the preferred imaging for diagnosis and treatment planning [7]. CBCT was introduced into the US market in the early 2000s and early adopters of this trend have now accumulated databases of serial craniofacial CBCT scans in healthy individuals. One advantage of utilizing a large CBCT database is the availability of multiple scans for each subject. This allows characterization of changes in the orbit of a single subject over time, in a longitudinal fashion. The complicating factor is the imperfect longitudinal character of the data. Scans are acquired based on clinical need rather than research purposes. For this reason, the resulting datasets are irregular and may consist of varying numbers of serial scans per individual and no consistency in the time elapsed between successive scans.

The purpose of this study was to measure changes in orbital volume from childhood to the late teenage years using serial CBCT scans of healthy individuals. To do this, we developed a protocol to segment and measure orbital volume on CBCT scans, and developed unique statistical tools to extract the maximum amount of information from the longitudinal dataset. Clinically, this information can be used to help determine optimal timing for pediatric orbital and lacrimal surgery where bone is removed or implants are placed, as well as in the management of the anopthalmic socket.

## Materials and methods

### Study population

This retrospective cohort study was approved at the exempt level by the Institutional Review Board at the University of Minnesota (STUDY00002026). All methods were performed in accordance with the relevant guidelines and regulations. All subjects included in the study or their legal guardians had provided informed consent for the use of orthodontic records for research purposes. The study cohort consisted of patients who had undergone orthodontic treatment at the University of Minnesota, and had pre- and post-treatment CBCT scans taken as part of orthodontic treatment. The radiographic records of an orthodontic patient typically consist of pre-, post-, and, occasionally, mid-treatment scans, used for diagnosis and treatment planning and outcome evaluation. This ensured the availability of a large database of CBCT scans from healthy individuals regardless of their craniofacial characteristics or the details of the treatment they received.

In order for a subject to be included, all of the following inclusion criteria had to be met: (1) The subject is Caucasian, (2) Age between 0 and 18 years on the day the initial scan was acquired, (3) At least two existing full field-of-view CBCT scans, and (4) All CBCT scans acquired on the same scanner with identical acquisition parameters to ensure consistent image quality across timepoints. Subjects were excluded if they met one or more of the following criteria: (1) History of craniofacial trauma, surgery, or pathology, (2) Craniofacial anomalies (e.g. cleft lip/palate), (3) Interval between successive scans less than 12 months, (4) Treatment with orthopedic appliances including headgear or functional appliances, (5) Previous or planned orthognathic surgery, (6) CBCT scans corrupted by motion artifact or poor image quality. The final study population consisted of 65 patients (24 male, 41 female), ages 6–18 years all of whom were treated with fixed orthodontic appliances (braces) between the years 2012 and 2017. The sex and age distribution are summarized in Table 1. Three patients had 4 serial CBCT scans, 10 patients had 3 serial scans, and 52 patients had 2 serial scans. In total, our sample consisted of 146 CBCT scans imaging 292 orbits.

**Table 1:** Age and sex distribution of CBCT scans

*Characteristic*	*Number of Scans*
*Panel A: Distribution by Sex*	
Female	95
Male	51
*Panel B: Distribution by Age*	
Age 7	6
Age 8	18
Age 9	9
Age 10	6
Age 11	6
Age 12	27
Age 13	15
Age 14	19
Age 15	12
Age 16	13
Age 17	10
Age 18	5

All CBCT scans were full field-of-view (17 × 23 cm) scans acquired at 120 kV and 18.54 mAs with a pulsed scan time of 8.9 s using an iCAT Next Generation scanner (Imaging Sciences International, Hatfield, PA, USA). The scan data were reconstructed with a voxel size of 0.3 mm^3^. All scans were fully deidentified prior to use in this study.

### Data collection

Data collection was performed using digital imaging and communications in medicine (DICOM) volumes. A total of three software programs were used at different stages of our workflow: Dolphin Imaging software (version 11.7; Dolphin Imaging & Management Solutions; Chatsworth, CA, USA), for orientation and anonymization of each scan, 3D Slicer (version 4.10.1, www.slicer.org), for segmenting the orbital bones and constructing artificial boundaries to turn each orbit into a closed object, and SmartPaint (version 1.5.1, http://www.cb.uu.se/~filip/SmartPaint/), for final segmentation of the orbital cavity and volume measurement. All steps of data collection were performed by a single operator (E.A.S.).

A detailed, step-by-step description of the protocol is provided in the Supplemental Materials. In brief, all scans were oriented using Dolphin Imaging Software. Scans were then imported into 3D Slicer, where they were cropped into right and left orbits. To transform the orbit into a closed space, we developed rules for establishing artificial boundaries where openings exist (Table 2). A virtual surface was generated for the anterior boundary in several steps using various Slicer modules: First, orbital bones were segmented using hysteresis thresholding and the orbital rim was manually outlined with fiducial markers in the Segment Editor. Next, the resulting markup list was used to generate a 3D surface using Delaunay Triangulation in the Markups-to-Model module. Finally, the ‘Volume crop with model’ module was used to set the intensity of all voxels contained within the 3D mesh model to zero.

**Table 2:** Openings of the orbital cavity and rules for delineating artificial boundaries

*Opening*	*Rule for generating boundary*
Anterior aperture	3D surface generated from fiducial landmarks placed along the crest of the orbital rim.
Optic canal	Cropping – the posterior border of the clipping box was adjusted to coincide with the intersection of the anterior limb of the optic strut and the optic canal, as viewed in three orthogonal, slice-based views.
Inferior orbital fissure	Manually delineated - convex path of closing generated by approximating the contour of adjacent bones. Approximated using a 2D interface in SmartPaint.
Superior orbital fissure	Manually delineated – convex path of closing generated by approximating the contour of adjacent bones. Approximated using a 2D interface in SmartPaint.
Nasolacrimal canal	Manually delineated – convex path of closing generated by approximating the contour of adjacent bones. Approximated using a 2D interface in SmartPaint.
Infraorbital canal	Roof of infraorbital canal provided anatomical boundary + manual delineation of posterior entrance
Supraorbital canal	Manually delineated – convex path of closing generated by approximating the contour of adjacent bones. Approximated using a 2D interface in SmartPaint.

**Fig. 1:**
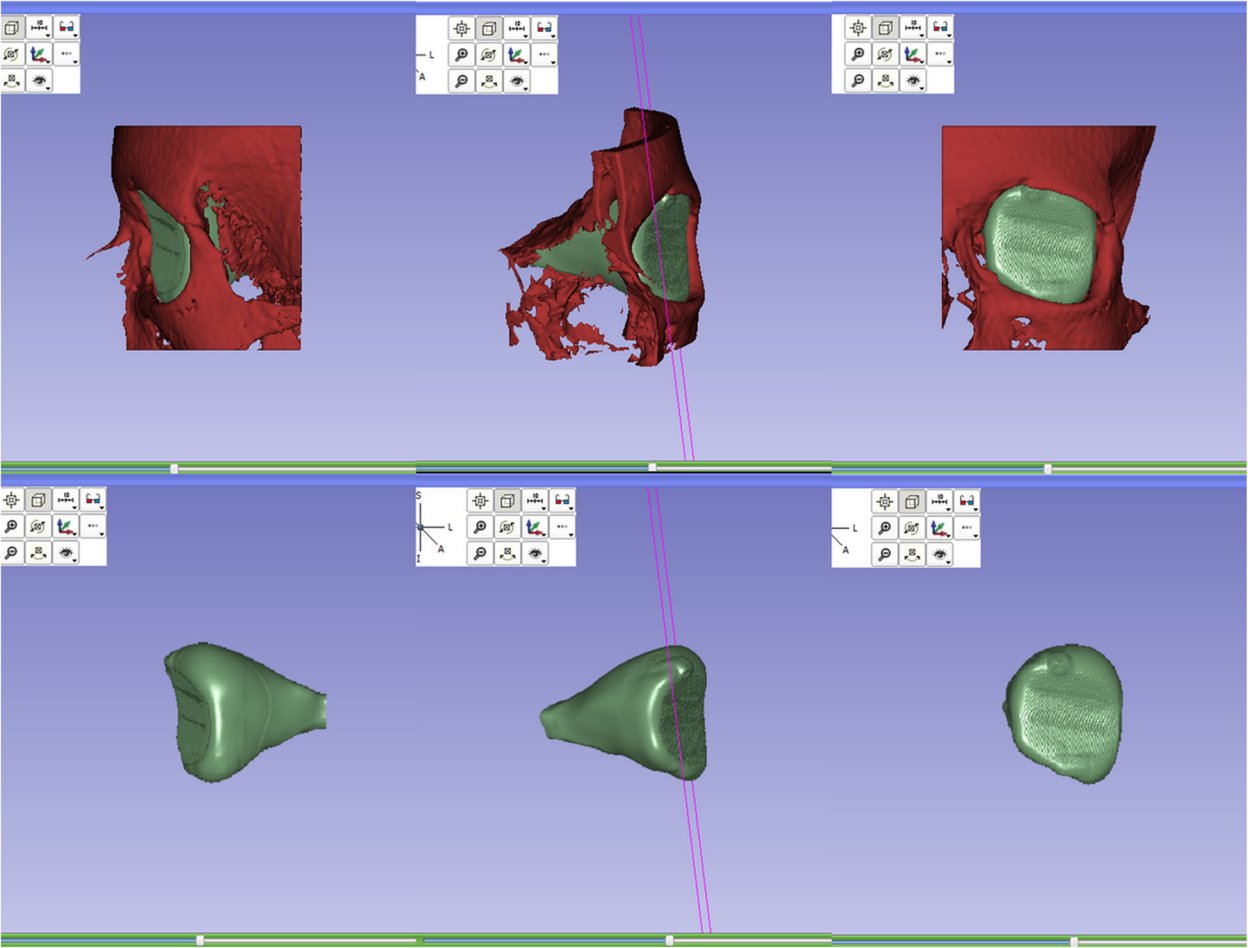
3D renderings of the segmented orbital cavity (green) and orbital bones (red), viewed in 3D Slicer. Lateral, medial, and frontal views

Segmentation of the orbital cavity and volume measurement was performed using SmartPaint. This software allows users to manually “paint” areas of an image using a 3D brush that, instead of an indiscriminate flood fill, selectively labels voxels according to Euclidean distance to the midpoint of the brush and the intensity values of the image. The remaining artificial boundaries were manually delineated by approximating a convex path between adjacent bones. In other words, the boundaries were edited until the segmentation was a smooth continuation of the orbit’s natural contours as viewed from all three orthogonal perspectives. Once the segmentation was completed, the SmartPaint software automatically calculated the volume by counting the number of labeled voxels. A 3D rendering of the result is shown in Fig. 1.

### Replicability study

To evaluate the reproducibility of measurements, we evaluated both intra-operator and inter-operator reliability. To assess intra-operator reliability, CBCT scans of a randomly selected subset of 10% (30 orbits) were re-analyzed by a single operator (E.A.S.), who repeated all steps of the data collection scheme after a four-week washout period. To evaluate inter-operator reliability, a second operator (C.S.H.) completed the measurements. The second operator was first calibrated with a two-day session using training materials (i.e. a step-by-step guide describing the data collection protocol) and by performing the protocol on 10 orbits not used elsewhere in the study. A four-week washout period followed. Then, both operators repeated all data collection steps on 30 randomly selected orbits. Both operators worked independently. Operators could reference the training materials while performing replications. Intraclass and interclass correlation coefficients were calculated to quantify the reproducibility of this protocol.

### Statistical analysis

All statistical analyses were conducted using Stata (version 14.2; StataCorp; College Station, TX, USA). To address the challenges of estimating orbital growth from an unbalanced panel of scans, we adapted a technique from the real estate finance literature. Specifically, we constructed a “Weighted Repeat Scan” (WRS) index. From a statistical perspective, our approach is identical to that described in Case & Shiller [8], which formed the basis for the well-known Case-Shiller House Price index. The approach relies on pairs of observations (i.e., scans) to estimate the growth rate between scans.

We began by assigning each scan an age, based on the age of the patient at the time of the scan. To do so, we divided the age of the patient in years by 365.25 and rounded the quotient to the nearest integer. For example, a scan of a patient aged 10 years and 7 months would be classified as the scan of an 11 year old. Because of thin data at the maximum of the age range, we reclassified the three scans of 19 year olds with the scans of the 18 year olds. We also averaged the volume measurements of left and right orbits for each patient at each point in time. The volume measure we employed is therefore the average of the measured volume of the patient’s left and right orbits.

For any two consecutive scans of the same individual *i*, we computed

1 $$growt{h_{i,k,j}}{\text{ }}={\text{ }}\ln (volum{e_{i,k}}){\text{ }}-{\text{ }}\ln (volum{e_{i,j}})$$

where $volum{e_{i,k}}$ is the volume of individual *i*’s orbit at age *k* in mm^3, $volum{e_{i,j}}$ is the volume of individual *i*’s orbit at age *j*, and *k* > *j*.

The WRS index was constructed using a three-step process. In the first step, we estimated the regression

2 $$growt{h_{i,k,j}}=\sum\limits_{{l \ne 12}}^{{}} {{\gamma _j}{D_{i,l}}} +{\varepsilon _{i,j,k}}$$

using an ordinary least squares (OLS) regression, where ${D_{i,l}}$ can take one of three variables: 1 if *k* = 1, −1 if *j* = 1, and 0 otherwise.

In the second step, we took the residuals from the first step, squared them, and estimated the OLS regression

3 $$\varepsilon _{{i,j,k}}^{2}=\alpha +\beta (k - j)+{\epsilon _{i,j,k}}$$

We then re-estimated the first regression using a weighted least squared (WLS) regression, where the square root of the fitted values from the second regression –$\sqrt {\hat {\varepsilon }_{{i,j,k}}^{2}}$ – are the regression weights. A WRS index produces a log growth index, which can then be converted into levels by exponentiating and multiplying by the average volume in the base year. Any year can be chosen as the base year for the purposes of the empirical analysis. We selected age 12 years because it was near the middle of the distribution, both in terms of ages and number of scans.

We tested for statistical significance of the coefficients estimated in the third step of the analysis using a two-sided t-test, implicitly testing whether the coefficient was different from that of the base year. The coefficients and standard errors are summarized in Table 3. Standard errors are clustered by patient. Clustering the standard errors in this way accounts for the possibility that the errors may be correlated across the scans of a single patient.

**Table 3:** Coefficient Estimates

	*Coefficient estimate*		
***Age***	***Pooled***	***Female***	***Male***
age 7	-0.0519*	-0.0609**	-0.0586
	(0.0219)	(0.0209)	(0.0297)
age 8	-0.103***	-0.0908***	-0.136***
	(0.0124)	(0.0149)	(0.0223)
age 9	-0.0678***	-0.0522**	-0.0686**
	(0.0143)	(0.0156)	(0.0235)
age 10	-0.0336	0.0180	-0.0724
	(0.0275)	(0.0259)	(0.0490)
age 11	-0.0489**	-0.0156	-0.0927**
	(0.0145)	(0.0198)	(0.0321)
age 12	---	---	---
[base year]			
age 13	-0.00591	0.0341	-0.0510
	(0.0159)	(0.0179)	(0.0256)
age 14	0.0457***	0.0439**	0.0529
	(0.0131)	(0.0138)	(0.0357)
age 15	0.0661***	0.0621**	0.0559***
	(0.0127)	(0.0191)	(0.0130)
age 16	0.0691***	0.0609**	0.0457
	(0.0138)	(0.0176)	(0.0284)
age 17	0.0784***	0.0650***	0.117***
	(0.0178)	(0.0175)	(0.0228)
age 18	0.0820***	0.0712**	0.106***
	(0.0149)	(0.0256)	(0.0273)
Observations	81	54	27
R-squared	0.756	0.770	0.866

Standard errors clustered by patient in parentheses. Statistical significance from a two-sided t-test is indicated as follows: *p<0.05, **p<0.01, ***p<0.001

Finally, a dependent t-test was used to analyze the difference between the average volume of the right and left orbits.

## Results

**Fig. 2:**
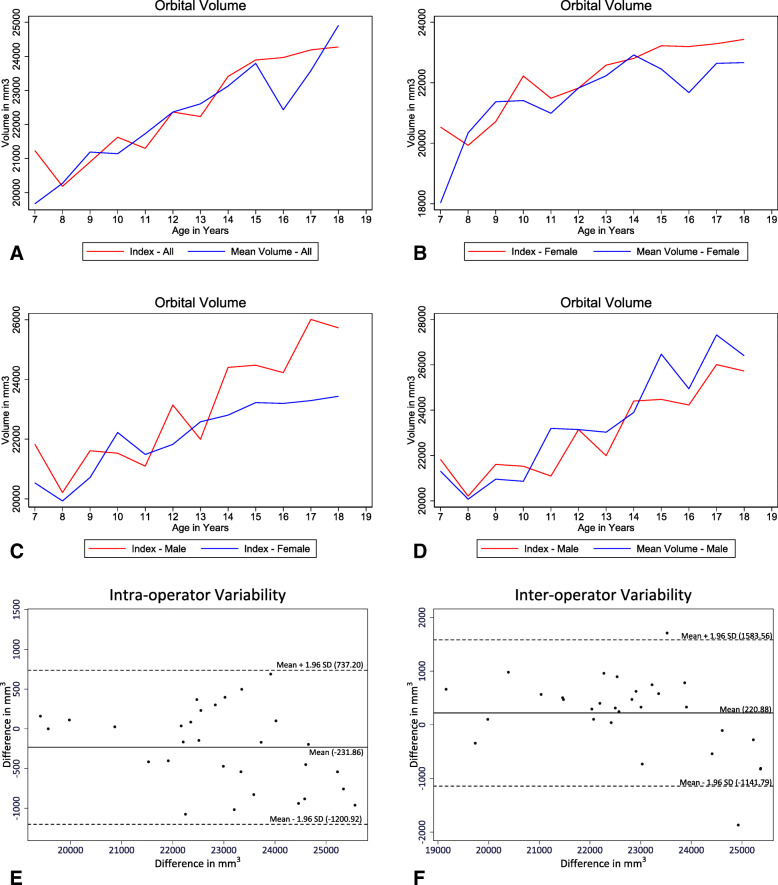
Changes in orbital volume as a function of age for the various subgroups and replications. Age 12 is the baseline year. **A** Simple Average vs. Index (pooling males and females, right and left orbits). **B** Simple average vs. index, females only (pooling right and left orbits). **C** Simple average vs. index, males only (pooling right and left orbits). **D** Index. Males and females only (pooling right and left orbits). **E** Bland-Altman plot showing the differences in repeat volume measurements on 30 orbits by a single operator. **F** Bland-Altman plot showing the differences in volume measurements on 30 orbits by two operators

The changes in orbital volume over the course of growth and development are shown in Fig. 2. Figure 2 A shows orbital volume plotted against age, pooling measurements from males and females and right and left orbits. Figure 2B and D show orbital volume plotted against age for females and males, respectively, and Fig. 2 C shows changes in orbital volume plotted against age for males and females. In general, orbital volume increased until the late teen years, with an approximate growth rate of 1–2% per year. The index for the male subsample was less smooth than that of the female subsample, which may be due in part to the smaller number of scans. Figure 2 C illustrates the fact that for both sexes, the orbital volume continued to increase well into the teens, with the increase more pronounced among males.

Results of the replicability study are depicted in the Bland-Altman plots shown in Fig. 2E F. The intraclass correlation coefficient was 0.945. The interclass correlation coefficient was 0.909.

In both the pooled group and in each of the male and female subsamples, the coefficient estimates for 8 year olds are smaller than zero, indicating that the average volume among these groups is smaller than those of 12 year olds (the base year), with the difference being statistically distinguishable (*P*<0.001) from zero at the 99.9% level (Table 3). Similarly, in all three columns, the coefficient estimates for 17 year olds is larger than zero, indicating that the average volume among these groups are larger than those of the 12 year olds. Here again, for all three groups (pooled, females and males), the coefficients are statistically distinguishable (*P*<0.01) from zero at the 99.9% level.

A statistically significant difference was found between the right and left orbital volumes, with the average volume of the right orbit approximately 0.5% larger than the left orbit (*p*<0.05).

## Discussion

This study aimed at generating findings with direct applicability to surgeons placing orbital implants. Proper reconstruction of orbital volume is critical in the management of the anopthalmic socket [9, 10]. For this reason, we chose to measure orbital volume as the single outcome since it is ultimately the variable of greatest clinical interest. The results suggest that changes in orbital volume continue until the late teen years, with an approximate growth rate of 1–2% per year. The finding that the growth rate is slow does not mean that these results are insignificant. Over years, even gradual increases can result in clinically relevant changes that may impact the long-term outcome of eye replacement procedures. The fact that orbital volume was found to increase into the late teen years challenges conventional notions of “maturity” and suggests that we may not yet know its ultimate endpoint. Our results corroborate findings of other long-term studies suggesting that orbital growth does not, as is often taught, end after adolescence, but instead slows to a low basal rate. Many of these studies assessed differences in bony orbital measurements between young and old adults and demonstrated continued growth and remodeling of the craniofacial skeleton throughout adulthood [11–13]. These bony changes manifest as a clockwise angular rotation of the bony orbit, with the forehead moving anteriorly and inferiorly and the midface moving posteriorly and superiorly. Overall, results from prior studies suggest that periods of bony remodeling persist throughout adulthood and may contribute to continued changes in orbital volume during adulthood [11–13].

Interestingly, the volume of the right orbit was on average about 0.5% larger than the volume of the left orbit (*P*<0.05). This finding is in contrast with previous studies, notably Bentley et al. [5] and Escaravage and Dutton [6]. Failing to find a significant difference, the aforementioned authors proceeded to use averaged data from right and left orbits to develop their growth curves. Both studies used an independent t-test to test for significance between right and left sides. Implicitly, their approach tested whether the difference between the *average* right orbit and the *average* left orbit was zero. In contrast, we tested whether the *average difference* between right orbit and left orbit was zero. In the context of an individual patient, we believe that this within-individual test is more appropriate. We also considered the ratio of volumes between right and left orbit rather than the simple difference. In fact, we reanalyzed the raw data provided in Bentley et al. [5] using our approach and found that the volume of the right orbit was larger than that of the left orbit. Specifically, we found that the mean difference in volume between an individual’s right orbit and left orbit is positive (*P*<0.01), and the ratio between the volume of an individual’s right orbit and that individual’s left orbit is greater than one (*P*<0.01). In both cases, this difference persists even when controlling for sex in an OLS regression specification with heteroskedasticity robust standard errors (*P*<0.01 and *P*<0.05, respectively). Since these studies were purely cross-sectional and thus relied on differences in average orbital volume to model orbital growth, this finding may have some relevance.

To our knowledge, this is the first study to use CBCT datasets to study age-related changes in orbital volume. Multiple challenges had to be overcome to generate interpretable data for this study. Orbital anatomy, with its thin bones and numerous openings with undefined boundaries, low growth rates, and image noise meant that great precision was needed to confidently detect very small changes in orbital volume. Following the example of numerous studies, [14–18] we used manual segmentation to segment and measure orbital volume. The major difference between our segmentation protocol and others is in how the orbital boundaries are defined. We developed a scheme to transform each orbit into an enclosed space with well-defined boundaries at all points in space. This involved generating a virtual barrier to seal the orbital aperture. Our method for delimiting the orbital entrance by constructing a boundary based on anatomical landmarks is most similar to Jansen et al., 2016 [16]. Where virtual boundaries could not be created due to an inability to accurately segment the thin orbital bones, rules were made to guide users in manually delineating the limits of the orbital cavity.

There is a large body of literature describing various protocols for measuring orbital volume with conventional CT [14–25] and MRI [5, 26]. While CBCT is capable of obtaining submillimeter resolution with isotropic voxels with far lower doses than conventional CT, it also posed unique challenges. These challenges – which include the lack of correspondence between grayscale values and actual Hounsfield Units and poorer image quality resulting from scatter artifacts and undersampling – precluded many of the time-saving automation steps used in protocols developed for conventional CT. These challenges cost us time rather than accuracy, since the fine resolution of CBCT images substantially minimizes error due to partial volume effects.

The fact that our data collection protocol included many steps and multiple software programs resulted in a potential for error. Being aware of this, we repeated all of the steps in our protocol and used a large sample when calculating intra-operator and inter-operator reliability. Since manual segmentation by knowledgeable, experienced operators is already considered by many authors to be the gold standard, [16, 27] a ‘validation’ step was deemed unnecessary. Although all measurements used for analysis were made by a single operator, we assessed inter-operator reproducibility to establish credibility of our new protocol. Following the approach of Regensburg et al., 2008 and Jansen et al., 2016, we evaluated inter-operator reliability using two operators [16, 26]. Despite the fact that it has many steps and uses multiple software programs, the inter- and intra-operator reliability were very high.

In the study of growth, longitudinal datasets are generally recognized as superior to cross-sectional data. Longitudinal data provide insight into individual variability of growth, and thus provides higher quality information from which growth can be studied and modeled. However, the considerable time and effort spent collecting longitudinal data is squandered if standard cross-sectional statistics are used to analyze the dataset. As noted by Schneiderman [28], the widespread use of longitudinal standards derived from conventional least-squares statistical methods (as in Riolo et al. [29]; Boersma et al. [30]; Behrents [31]) gives a spurious impression of low variability and tends to exaggerate the significance of treatment effects [29–31]. Our sample contained varying numbers of serial observations, with no consistency in terms of duration between successive scans or the number of scans per individual. Therefore, a different approach was needed to preserve the longitudinal character and extract the maximum amount of information regarding orbital growth.

The use of the WRS approach represents a second methodological contribution of this paper. While the WRS methodology was adapted from a very different context – the creation of a real estate index – from a statistical perspective, the underlying problems in our dataset mirror those for which the methodology was developed. In both contexts, observations appear at irregular intervals: houses sell only periodically and CBCT scans are taken when necessary for clinical purposes rather than at regular intervals. In both contexts, there is substantial heterogeneity across individuals which must be controlled for: each house has unique characteristics, just as each patient’s skull is distinct. The methodology controls for time-invariant features of individuals by relying on changes *within* individual over time, rather than looking *across* individuals. Finally, what in financial economics is termed a “return” is mathematically equivalent to a growth rate. Given all these features, the WRS methodology is well suited for use in this context.

The described methodology for utilization of CBCT databases available through dental departments can help orbital and lacrimal surgeons better understand the growth of the pediatric orbit. This can, with future studies, help guide timing and implant selection for anophthalmic socket procedures, orbital trauma reconstruction, and lacrimal surgery. For our study, we chose to measure a single outcome (volume), but future studies can focus on finer details and patterns of orbital growth. In particular, determining the mechanism of childhood orbital volume expansion, and whether it involves bone deposition or resorption, would be helpful in order to evaluate the similarities and differences between orbital changes during childhood and the bony remodeling that occurs during adulthood.

## Conclusions

Orbital volume increases by 1–2% per year throughout childhood continuing until the late teenage years. This annual increase is large enough to be clinically relevant as it may lead to less-than-optimal long term surgical outcomes when reconstructive surgery for the pediatric anophthalmic socket is required.

## Supplementary information

**Additional file 1.**

## Data Availability

The datasets used and/or analyzed during the current study are available from the corresponding author on reasonable request.
