# Supplementary material for: Orbital volume changes during growth and development in human children assessed using cone beam computed tomography
Source: Head Face Med. 2022 Feb 28;18:8. doi: 10.1186/s13005-022-00310-9 (PMC8883635; doi:10.1186/s13005-022-00310-9)
Supplement: Supplementary file 1 — Additional file 1. [file 13005_2022_310_MOESM1_ESM.docx]

**Supplementary Materials**

This appendix details the protocol used in the study “Orbital volume changes during growth and development in human children assessed using cone beam computed tomography.” An overview of this protocol is depicted in Supplementary Figure S1. Step numbers indicated in each box correspond to the section numbers in the text below.

(i) All scans were oriented (Supplementary Figure S2), anonymized, and exported using Dolphin Imaging Software. Skulls were oriented to Frankfort Horizontal to ensure consistent cropping of left and right orbits into separate volumes. Since the orbit contains numerous openings, measuring orbital volume depends on how one defines its limits. Once all boundaries are defined, the problem reduces to one of labeling each voxel as belonging to ‘inside’ or ‘outside’ the orbital cavity and counting the total number of voxels with the ‘inside’ label. Table 1 identifies all openings in the orbital cavity and describes how, for each opening, an artificial boundary was established.

(ii) Each scan was then imported into the open-source software 3D Slicer, where the following workflow was implemented: Each volume was cropped to separate right and left orbits using the ‘Crop Volume’ module. The posterior border of the clipping box was adjusted to coincide with the point of intersection of the ascending ramus of the optic strut with the floor of the optic canal (Supplementary Figure S3). Each orbit was saved as a separate volume and assigned a randomly-generated filename (www.random.org) to ensure blinding. This step served two purposes: (1) restricting the field of view to exclude irrelevant areas (e.g. the jaws) to conserve memory and expedite all subsequent steps in this workflow, (2) establishing a posterior limit for the orbital cavity and restricting the measurement to structures anterior to the optic canal.

(iii) Next, the orbital bones were segmented by hysteresis thresholding in 3D slicer’s ‘Segment Editor’ module. This allowed accurate visualization of the orbital rim in 3D. To avoid confounding expansion of the orbit with protrusion of the globe, which has been shown to increase with age, the orbit's anterior opening was closed in order to exclude the globe from measurement. For this, the orbital rim was marked by placing fiducial markers in the 3D view of the segmentation. Starting at the frontozygomatic suture, fiducial markers were placed every 5mm along the crest of the orbital rim. After interacting with the 3D segmentation to produce the initial markup, the position of each fiducial marker was fine-tuned to coincide with the crest of the orbital rim in each of the three orthogonal views. The orbital crest, an elevation on the orbital rim, is well defined and distinct except for its medial wall. Here, fiducial markers were placed along the anterior lacrimal crest. An example of a final markup is shown in Supplementary Figure S4.

Next, a 3D mesh model was generated from these fiducials using the ‘Markups-to-Model’ extension in 3D Slicer. This extension uses the fiducial markers as inputs and generates a 3D surface using Delaunay Triangulation. Finally, 3D Slicer’s ‘Volume crop with model’ module was used to ‘zero’ all of the voxels contained within the 3D mesh model. An example of the final result is shown in Supplementary Figure S5. The resulting volume is then saved as a .vtk file. The boundaries of the orbit are now established and it is ready for segmentation.

(iv) Lastly, segmentation of the orbital cavity and volume measurement was performed using SmartPaint v1.5.1 (http://www.cb.uu.se/~filip/SmartPaint/). SmartPaint allows users to manually “paint” areas of an image using a 3D brush that, instead of an indiscriminate flood fill, selectively labels voxels according to Euclidean distance to the midpoint of the brush and the intensity values of the image. The relative sensitivity of the brush to Euclidean distance from the brush’s center and image content (intensity values) can be adjusted by the operator. Thus, the user swipes the brush, and the ‘paint’ automatically adapts itself to the anatomical and virtual boundaries in 3D. Since the brush tends to produce segmentations with irregular edges when used on noisy images, the software also incorporates a smoothing operation which can be applied to the entire segmentation. The remaining boundaries were manually delineated by approximating a convex path between adjacent bones. In other words, the user edited these boundaries until the segmentation was a smooth continuation of the orbit’s natural contours as viewed from all three perspectives. Examples are shown in Supplementary Figure S6. Once the segmentation was completed, the SmartPaint software automatically calculated the volume by counting the number of labeled voxels.


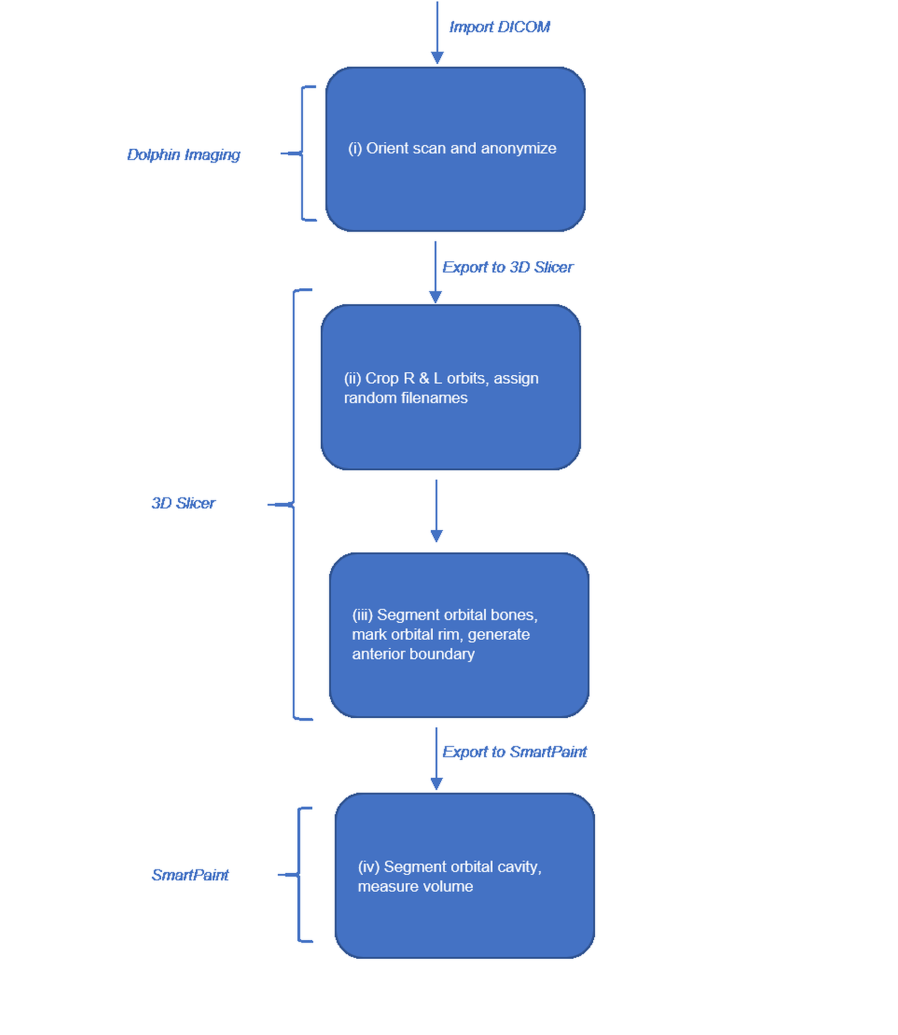


**Supplementary Figure S1.** Overview of data collection. Step numbers indicated in each box correspond to the section numbers in the text. Each step was performed for all scans before proceeding to the next.


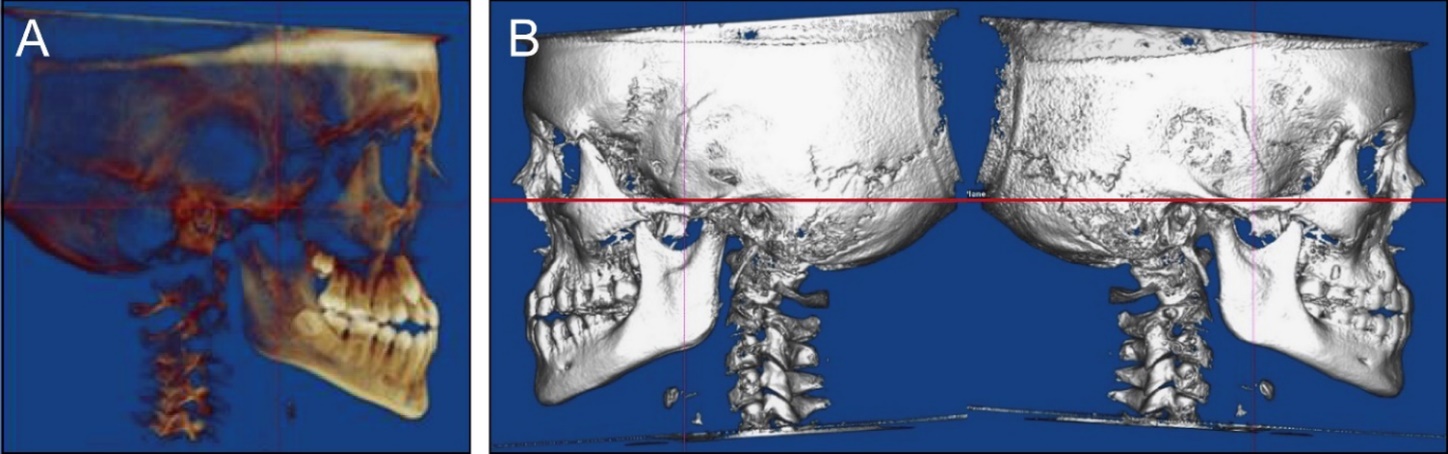


**Supplementary Figure S2.** Orientation of scans in Dolphin Imaging software. **A.** A translucent view was used to superimpose the orbital rims and the zygomatic processes of the maxilla. **B.** Solid left and right lateral views were used to orient the skull to Frankfort horizontal.


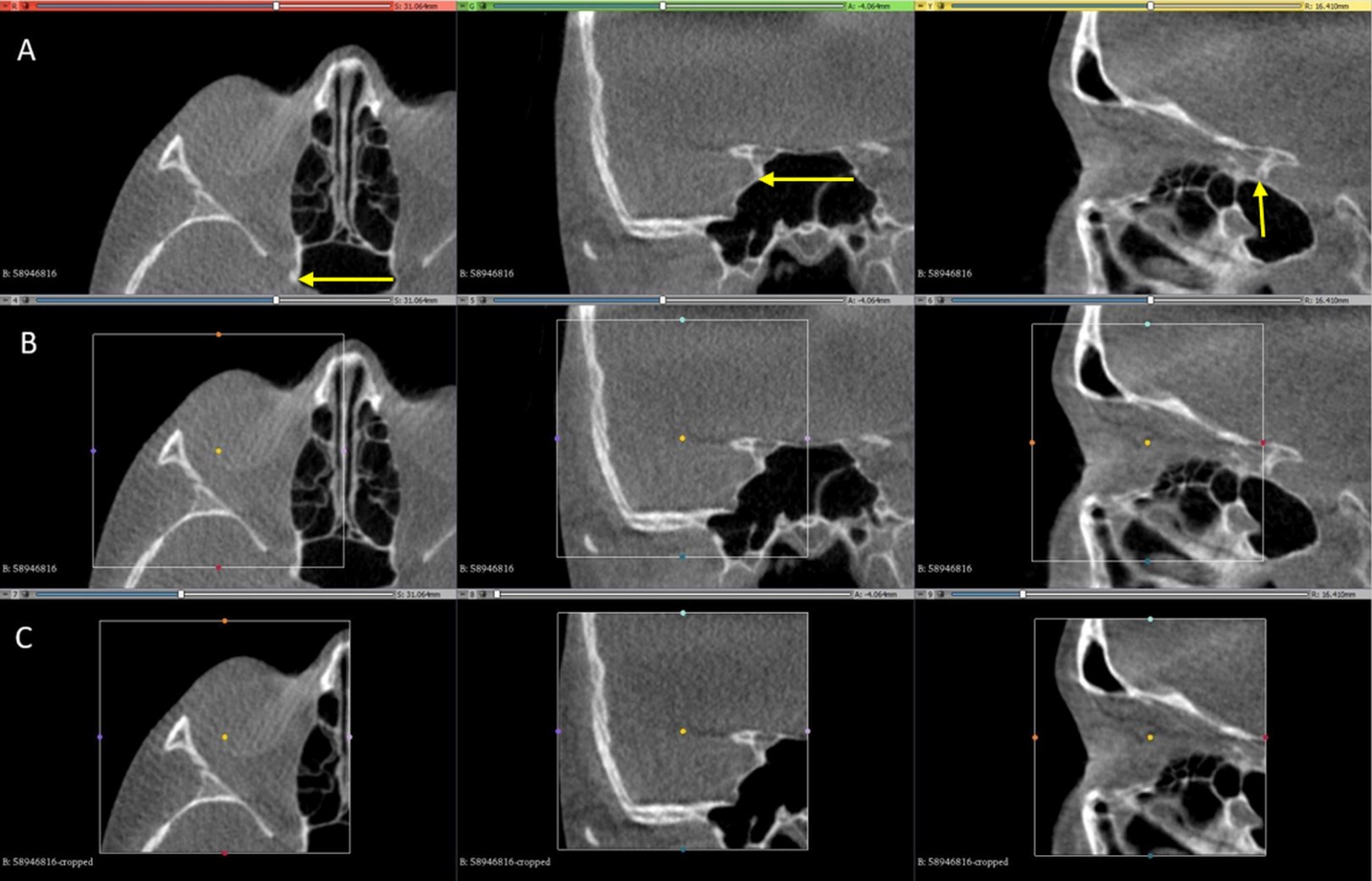


**Supplementary Figure S3.** **A.** The point of intersection of the ascending limb of the optic strut with the floor of the optic canal, as identified in sagittal (yz), coronal (xz), and axial (xy). The landmark was placed at the tip of the yellow arrow. The landmark was first located in the sagittal view, and coronal and axial views were then used to adjust and fine-tune its position. **B and C.** Each orbit was cropped using a 3D clipping box. The posterior border of the box was positioned so that it passed through the landmark.


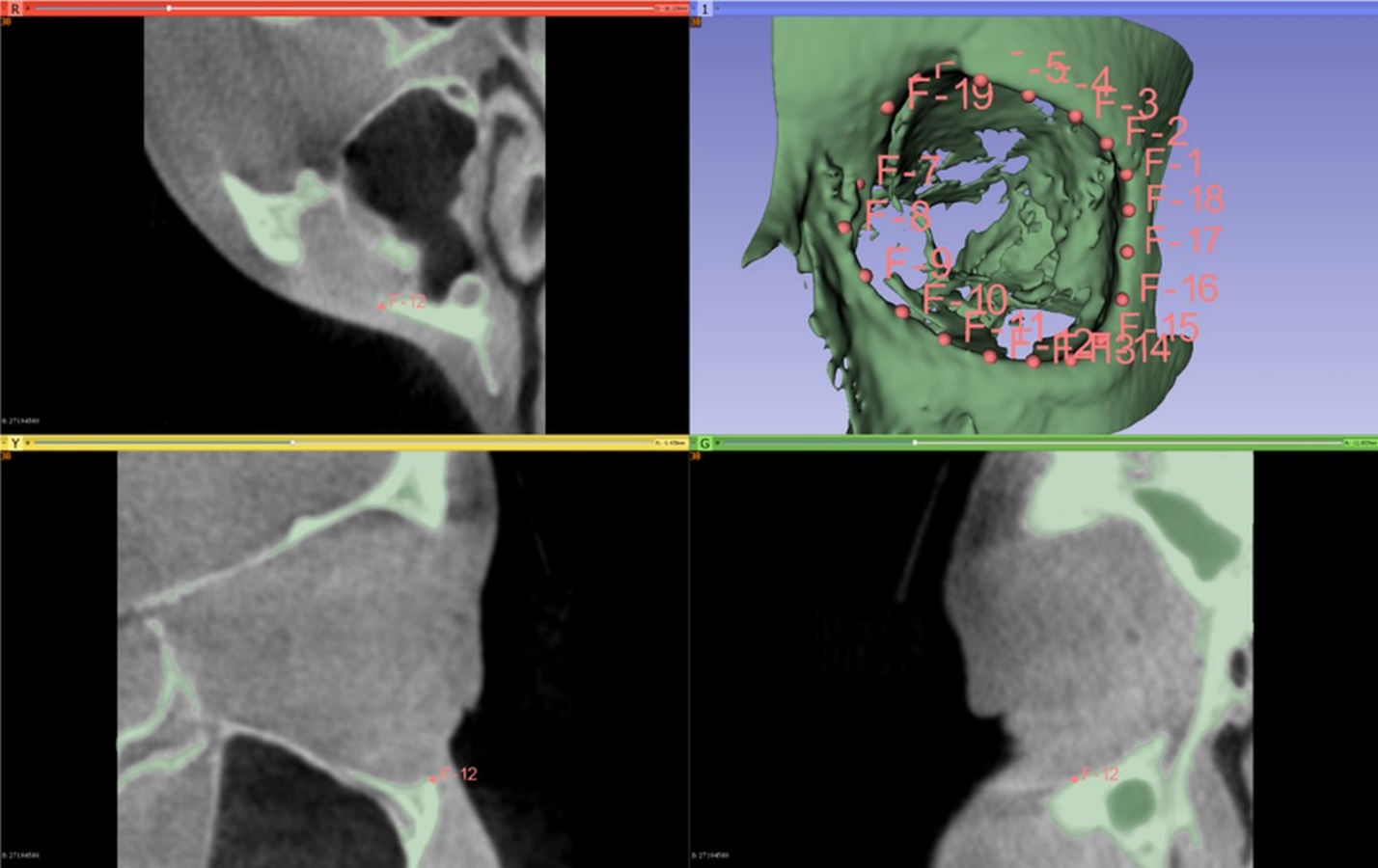


**Supplementary Figure S4.** Fiducial landmark placement. The initial markup was produced by interacting with the 3D rendering of the segmentation. Next, each point was adjusted to coincide with the crest of the orbital rim in the 2D slice views.


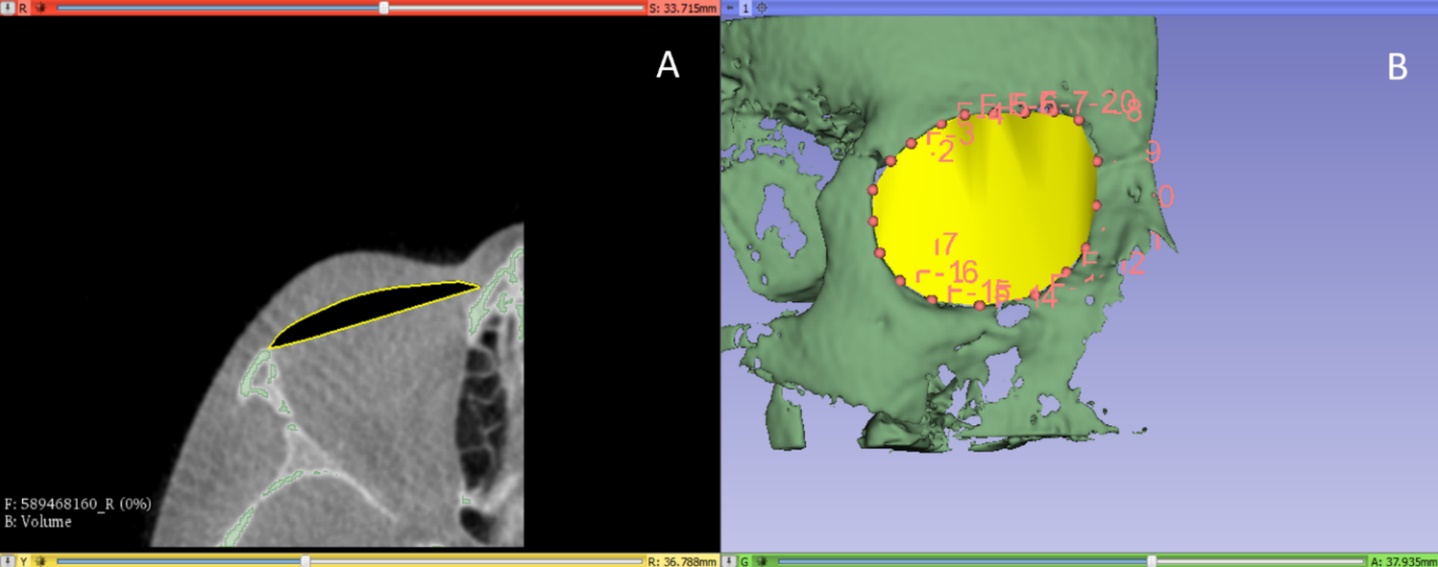


**Supplementary Figure S5.** Generation of anterior boundary **A.** Axial cross-section. **B.** 3D surface generated using the fiducial landmarks as inputs.


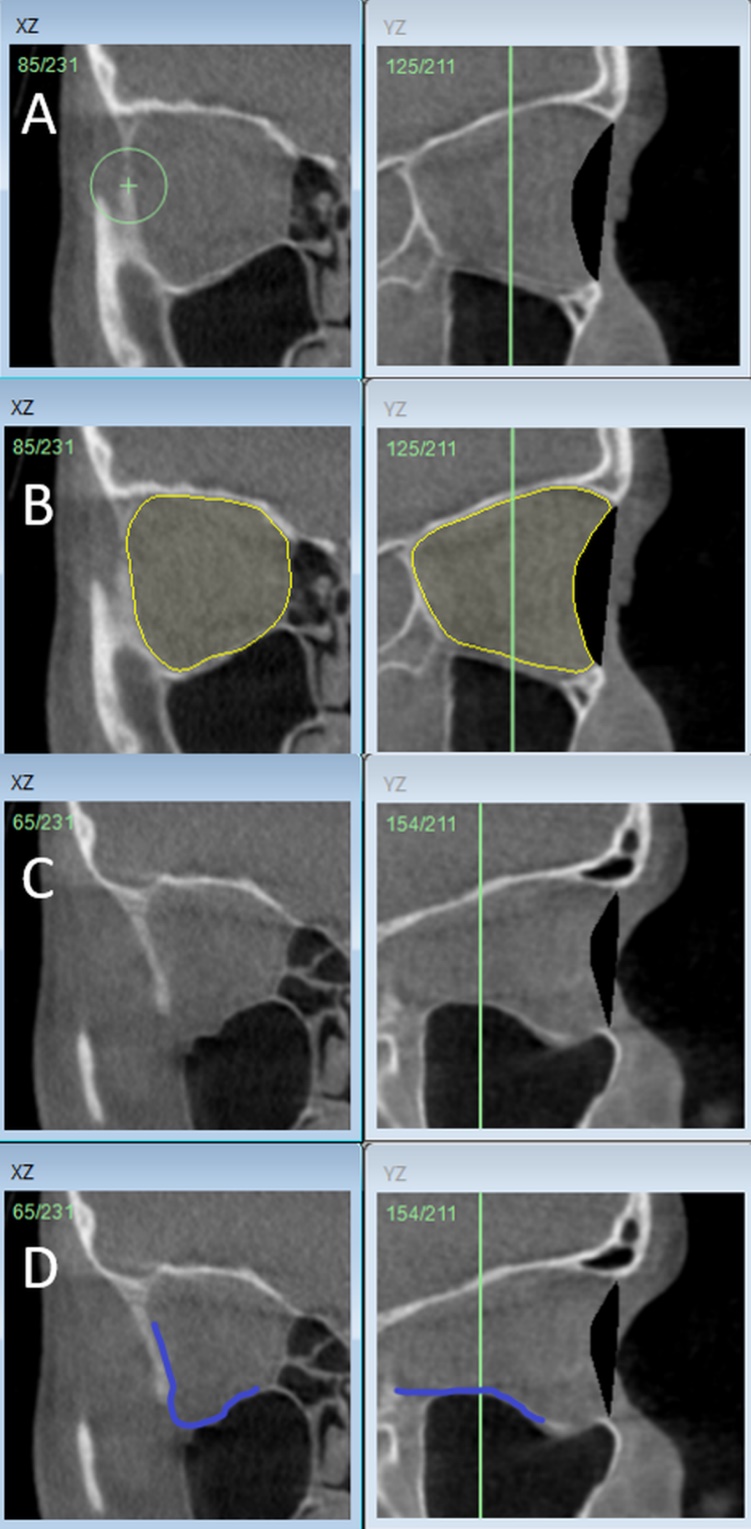


**Supplementary Figure S6.** Manual segmentation of the orbital cavity in SmartPaint. Users “paint” the orbital cavity using a selective 3D brush that takes account of voxel intensity and Euclidean distance from the brush’s center. **A and B.** Operators can adjust the brush’s sensitivity to either of these variables, resulting in a segmentation that readily adapts itself to anatomical and virtual boundaries**. C and D.** Where artificial boundaries are not established, users can manually delineate the limits of the orbital cavity by extending the contours of adjacent bones and approximating convex path of closure.
